# Genomic landscape and homologous recombination repair deficiency signature in stage I-III and de novo stage IV primary breast cancers

**DOI:** 10.1093/oncolo/oyaf089

**Published:** 2025-05-27

**Authors:** June E Jeon, Kuei-Ting Chen, Russell Madison, Alexa B Schrock, Ethan Sokol, Mia A Levy, Mariya Rozenblit, Richard S P Huang, Lajos Pusztai

**Affiliations:** Department of Internal Medicine, Greenwich Hospital, Greenwich, CT, USA; Foundation Medicine, Cambridge, MA, USA; Foundation Medicine, Cambridge, MA, USA; Foundation Medicine, Cambridge, MA, USA; Foundation Medicine, Cambridge, MA, USA; Foundation Medicine, Cambridge, MA, USA; Yale Cancer Center, Yale School of Medicine, New Haven, CT, USA; Foundation Medicine, Cambridge, MA, USA; Yale Cancer Center, Yale School of Medicine, New Haven, CT, USA

**Keywords:** genomic, breast cancer, recurrence, homologous recombination repair, targeted molecular therapy, homologous recombination deficiency signature

## Abstract

**Purpose:**

We compared genomic alterations and a homologous recombination deficiency (HRD) signature (HRDsig) in primary tumors from stage I-III to those in de novo stage IV breast cancers and from stage I-III cancers with early (<2 years after diagnosis) versus late (>2 years) recurrence.

**Methods:**

De-identified genomic and clinical data of primary breast cancers (stage I-III *N* = 910, stage IV *N* = 783) from the United States, the nationwide clinico-genomic database of Flatiron Health and Foundation Medicine were analyzed. Genomic results included the mutation status of 324 cancer-related genes and HRDsig, a DNA scar-based measure of HRD.

**Results:**

No significant differences were observed in the frequencies of genomic alterations across disease stages, or between stage I-III cancers with early versus late relapse. Overall, the most prevalent biomarkers were *PIK3CA* mutation and HRDsig positivity. HRDsig positivity was observed in 82% of germline or somatic g/s*BRCA1/2* or germline *PALB2 (gPALB2)* mutated cancers, 13.1% in cancers with other HR-repair (HRR) gene alterations (*ATM, BARD1, BRIP1, CDK12, CHEK1, CHEK2, FANCL, somatic PALB2 (sPALB2), RAD51B, RAD51C, RAD51D* and *RAD54L)*, and also in 16.5% of HRR wild-type cancers. HRDsig positivity was observed across receptor subtypes and was the highest in TNBC (30%), followed by ER+/HER2- cancers (17%), then HER2 + cancers (8.7%).

**Conclusions:**

Early-stage (I-III) and de novo stage IV breast cancers shared a similar prevalence of targetable genomic alterations and overall genomic landscape. HRDsig identified approximately 16% of breast cancers without g/s*BRCA*/*gPALB2* alteration that might potentially benefit from PARP inhibitors or platinum-based treatments and should be tested in future clinical studies.

**Implications for Practice**

Comprehensive genomic profiling data from cancer biopsies is used to guide treatment. Our study shows that primary tumors of stage I-III and stage IV breast cancers share similar targetable genetic alterations and similar overall genomic landscapes. We also found that a novel DNA scar-based biomarker of homologous recombination deficiency (HRDsig) is observed in 16% of breast cancers without g/s*BRCA*/*gPALB2* alteration, that raise the possibility of benefit from PARP inhibitors or platinum-based treatment. Future clinical trials are needed to validate the predictive value of HRDsig.

## Introduction

Over 300 000 new cases of breast cancer will be diagnosed in the United States in 2025 and more than 90% of newly diagnosed breast cancers are stage I-III disease; the rest are stage IV cancers, also called de novo metastatic breast cancer.^1^ Systemic therapies administered before and/or after surgery for stage I-III cancers are increasingly effective in eradicating micrometastatic disease. Consequently, metastatic recurrences of stage I-III breast cancers are steadily decreasing while the proportion of de novo stage IV cancers among metastatic breast cancers is increasing. Historically, about 20% of metastatic breast cancers were stage IV disease and the rest were recurrences; the contemporary proportion of stage IV cancers among all metastatic cancers is closer to 30%-40%.^2^ Whether the molecular features of primary tumors that present with distant metastases at diagnosis (ie, stage IV) differ from stage I-III breast cancers that may only harbor micrometastases is unknown. Next-generation sequencing technology has allowed for rapid DNA sequencing to generate patient-specific comprehensive genomic profiling (CGP) data from cancer biopsies to guide treatment. Currently, tumor alterations in *PIK3CA*, *AKT1, PTEN, ESR1*, *HER2*, *NTRK1/2/3*, *RET*, and germline or somatic alterations in *BRCA1/2* genes, germline *PALB2* (gPALB2) alterations, as well as microsatellite instability (MSI) and high tumor mutation burden (TMB) represent actionable biomarkers in breast cancer.^3^

Comprehensive genomic data is also used to identify genome-wide mutational patterns and to elucidate new targetable genomic alterations.^4^ For example, germline or somatic pathological mutations in *BRCA1* and *BRCA2* genes (g*BRCA1/2*) lead to impaired homologous recombination repair (HRR) that results in a unique mutation pattern or “DNA scar.” Homologous recombination plays a key role in the template-dependent repair of complex DNA damage, thus maintaining genomic stability.^5^ Cells with HR deficiency (HRD) are dependent on non-homologous DNA end joining for DNA repair through the poly(ADP-ribose) polymerase (PARP) catalytic activity. PARP inhibitors (olaparib and talazoparib) are FDA-approved therapies that are cytotoxic to tumors with HRD and have demonstrated efficacy as adjuvant therapy in *gBRCA1/2* positive breast cancer and as treatment of metastatic breast cancers with g*BRCA1/2* mutation.^6,7^ A Phase II clinical trial also demonstrated the clinical efficacy of olaparib in *gPALB2* and somatic *BRCA1/2 (sBRCA1/2)* mutant cancers.^8^ HRR is also involved in correcting DNA interstrand crosslinks that can be induced by platinum drugs (eg, carboplatin, cisplatin), therefore, *BRCA1* mutant metastatic breast cancers are also highly sensitive to these drugs.^9,10^ Several studies have shown that some tumors without *BRCA1* and *BRCA2* mutation also have a “BRCAness” phenotype indicated by genomic scars typical for HRD, that may be caused by promoter methylation and other forms of silencing of genes that mediate DNA repair. These cancers might also benefit from PARP inhibitors and platinum salts.^11,12^ Using CGP data, a machine learning algorithm, called HRDsig (Foundation Medicine), was developed to predict the HRD functional readout by analyzing genome-wide patterns in a broad set of copy number features.^13^

In this study, we compared genomic alterations in primary tumors from stage I-III (early-stage) to those from de novo stage IV (late-stage) breast cancers, and from stage I-III cancers with early (≤2 years after diagnosis) versus late (> 2 years) recurrence. We examined the prevalence of targetable gene alterations including PD-L1 protein expression, the overall genomic landscape of alterations in 324 cancer-related genes, and HRDsig across these groups.

## Materials and methods

### Patient selection and genomic data

We used the US nationwide de-identified Flatiron Health and Foundation Medicine Inc. (FMI) clinico-genomic database (CGDB; https://flatiron.com/real-world-evidence/clinico-genomic-database-cgdb). The CGDB includes patient-level structured and unstructured retrospective longitudinal clinical and genomic data from approximately 280 cancer clinics corresponding to 800 care sites in the United States. The clinical information is extracted from electronic health records using technology-enabled abstraction augmented by human curation, the genomic data is derived from the FMI CGP tests.^14^ Institutional Review Board approval of the study protocol was obtained prior to study conduct and included a waiver of informed consent based on the observational, non-interventional nature of the study.

**Figure 1.**
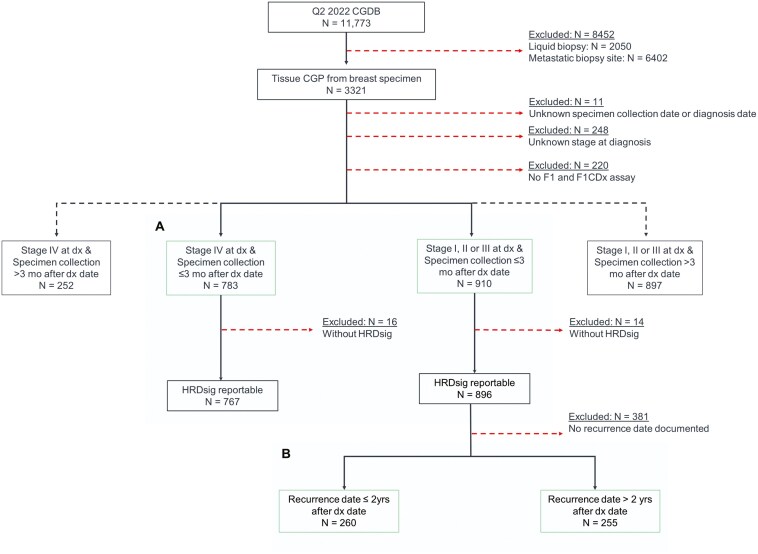
CONSORT diagram of patient selection. Subpanels indicate the 2 primary comparison cohorts. **(A)** stage I-III versus stage IV. **(B)** early versus late recurrence among stage I-III cancers. Abbreviations: Q2 2022 CGDB, comprehensive genomic database accessed on second quartile of year 2022; CGP, comprehensive genomic profiling; dx, diagnosis; mo, months; HRDsig, homologous recombination repair deficiency signature; yrs, years,

The Flatiron Health-FMI CGDB included 11 773 patients with breast cancer who had an FMI CGP assay performed between January 1st, 2011 to March 31st, 2022. Patients who had CGP of a liquid biopsy (*N* = 2050) or tissue biopsy of a metastatic site (*N* = 6402) were excluded from this analysis, as well as patients with unknown stages at diagnosis, unknown date of specimen collection, or incomplete follow-up information. Specimens collected for CGP more than 3 months after diagnosis were also excluded because these patients likely have received some neoadjuvant therapy before specimen collection that could alter genomic profiles.^15,16^ The final study cohort consisted of 1693 breast cancer patients with genomic results obtained from primary breast cancers within 3 months of diagnosis using the FoundationOne® (F1) or FoundationOne® CDx (F1CDx) assays (Figure 1). Stage was assigned using the American Joint Commission on Cancer version 7 recommendations, stage IV disease corresponds to *de novo* metastatic breast cancer. Stage I-III cancers were further subdivided into those with early and late recurrence, defined as metastatic recurrence ≤ 2 or >2 years from diagnosis, respectively.^17,18^

### Comprehensive genomic profiling

Genomic profiling was performed in a Clinical Laboratory Improvement Amendments-certified, CAP (College of American Pathologists)-accredited laboratory of Foundation Medicine Inc., (Cambridge, MA, USA) in the context of routine clinical care. Briefly, DNA was extracted from a total of 40 microns of FFPE sections and next-generation sequencing was performed on hybridization-captured, adaptor ligation-based cDNA libraries to a mean coverage depth of >550× for over 300 cancer-related genes plus selected introns from genes frequently rearranged in cancer, as previously described.^19^

Tumor mutational burden (TMB) was calculated by counting the number of synonymous and non-synonymous non-driver mutations across 0.8-1.2 megabase (Mb) and reported as mutations/Mb after computational germline variant filtering.^20^

Somatic, germline, and allelic status calls were determined using a computational zygosity prediction algorithm.^19^ Biallelic calls were made for instances involving (1) a deep deletion in the gene, (2) 2 or more pathogenic alterations in the same gene, or (3) a pathogenic short variant under loss of heterozygosity. Monoallelic alterations refer to single-short variant alterations identified as heterozygous. In cases where zygosity could not be reliably assessed (eg, for rearrangements or when the zygosity call was indeterminate), an allelic status of “unknown” was assigned.

A Homologous Recombination Deficiency (HRD) signature (HRDsig) was measured as previously described.^17^ Briefly, the HRDsig algorithm extracts copy number features from segmented copy number profiles which are used as inputs for machine learning. The model was trained on cases with biallelic *BRCA1/2* as a true positive and samples wild type for 14 HRR genes (*BRCA1, BRCA2, ATM, BARD1, BRIP1, CDK12, CHEK1, CHEK2, FANCL, PALB2, RAD51B, RAD51C, RAD51D* and *RAD54L*) as true negatives.

PD-L1 protein expression was assessed using the DAKO 22C3 assay and a combined positivity score (CPS) threshold of ≥10 defined PD-L1 positivity.

### Data analysis

We compared the frequency of clinicopathologic characteristics and HRD status between Stage I-III and Stage IV cancers, and between stage I-III cancers with early versus late recurrence, respectively using Fisher’s exact test for categorical variables (eg, tumor grade) and the Kruskal–Wallis test for continuous variables. *P*-values were adjusted for multiple comparisons using the Benjamini–Hochberg false discovery rate (FDR) method. Data analysis was conducted using the RStudio (R version 4.1.0).

## Results

### Targetable biomarkers in stage I-III and de novo stage IV cancers

Eight hundred and ninety-six cancers were stage I-III and 767 were stage IV. Patients with stage IV disease compared to stage I-III were slightly older at diagnosis (median age 57 vs 60, *P* = .01), more frequently had HR+/HER2- disease (52.8% vs 61.2%, *P* = .005); and less frequently were TNBC (36.4% vs 28.5%, *P* = .005). No statistically significant differences were found in HRDsig, MSI-H, predicted ancestry, PD-L1 protein expression, and HER2 status (Table 1).

**Table 1.** Study population and the clinicopathologic characteristics of specimens of stage I-III and de novo stage IV cancers.

Clinicopathologic characteristics	Stage I-III (*N* = 896)	Stage IV (*N* = 767)	*P* val (FDR)
Age at diagnosis			.01
Median (Q1, Q3)	57 (47, 67)	60 (50, 68)	
*HRDsig*			.58
positive	188 (21.0%)	177 (23.1%)	
negative	708 (79.0%)	590 (76.9%)	
*TMB*			.1
<10	865 (96.6%)	725 (94.5%)	
≥10	30 (3.4%)	42 (5.5%)	
N-Miss	1	0	
MSI-H			1.0
MSI I/L	894 (99.8%)	765 (99.7%)	
MSI-H	2 (0.2%)	2 (0.3%)	
*Receptor status*			
HER2+	95 (10.8%)	78 (10.3%)	.96
HR+/HER2-	466 (52.8%)	463 (61.2%)	.005
TNBC	321 (36.4%)	216 (28.5%)	.005
N-Miss	14	10	
*Predicted ancestry*			
AFR	128 (14.3%)	120 (15.6%)	.73
AMR	110 (12.3%)	67 (8.7%)	.07
EAS	18 (2.0%)	12 (1.6%)	.84
EUR	631 (70.4%)	560 (73.0%)	.55
SAS	9 (1.0%)	8 (1.0%)	1.00
*PD-L1 status (DAKO 22C3), CPS ≥ 10*			.96
Positive	14 (45.2%)	11 (39.3%)	
Negative	17 (54.8%)	17 (60.7%)	
N-Miss	865	739	

Abbreviations: *P* val, *P*-value; FDR, false discovery rate; Q1, 1st quartile; Q3, 3rd quartile; AFR, Africans; AMR, Admixed Americans; EAS, East Asians; EUR, Europeans; SAS, South Asians; PD-L1, programmed cell death ligand 1; CPS, combined positive score; N-Miss, data missing; TMB, tumor mutational burden; MSI-H, microsatellite instability-high; MSI I/L, microsatellite instability- indeterminate/low; HR, hormone receptor; HER2, human epidermal growth factor receptor 2; TNBC, triple-negative breast cancer; IDC, invasive ductal carcinoma; ILC, invasive lobular carcinoma.

**Figure 2.**
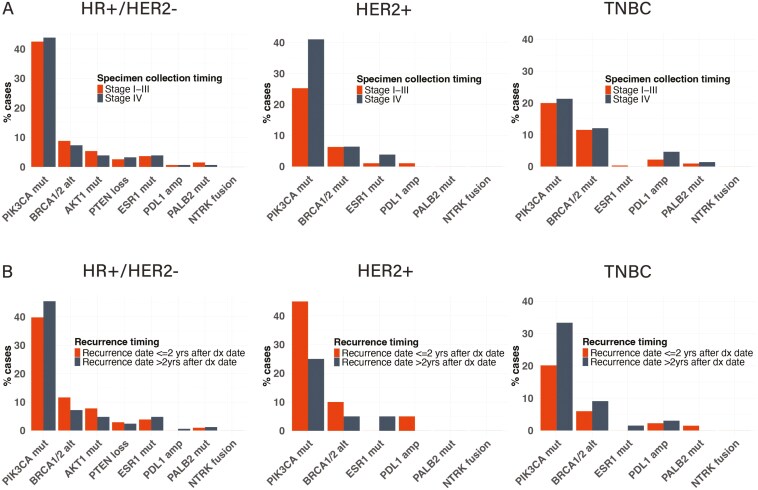
Prevalence of targetable gene alterations by hormone receptor status. **(A)** Comparison between stage I-III versus stage IV breast cancers. **(B)** Comparison between stage I-III cancers with early versus late recurrence. Abbreviations: HR, hormone receptor; HER2, human epidermal growth factor receptor 2; TNBC, triple-negative breast cancer; dx, diagnosis; *PIK3CA* mut, phosphatidylinositol-4,5-bisphosphate 3-kinase catalytic subunit alpha gene mutation; *BRCA 1/2* alt, breast cancer gene 1 and 2 alteration; *ESR1* mut, estrogen receptor alpha gene mutation; *PDL1* amp, programmed cell death ligand 1 gene amplification; *PALB2* mut, partner and localizer of *BRCA2* gene mutation; *NTRK* fusion, neurotrophic tyrosine receptor kinase gene fusion, *AKT1* mut, RAC-alpha serine/threonine-protein kinase gene mutation; *PTEN* loss, phosphatase and tensin homolog gene loss.

The prevalence of currently targetable alterations was compared after stratification by 3 receptor subtypes (HR+/HER2−, HER2+, TNBC). There were no significant differences in the frequencies of targetable alterations between stage I-III and stage IV cancers in any of the receptor subtypes (Figure 2A). Among HR+/HER2- stage I-III (*N* = 466) and stage IV (*N* = 463) breast cancers, the most frequent alterations were *PIK3CA* mutations (42.5% vs 43.8%, respectively); followed by *BRCA1*/*2* mutations (8.8% vs 7.3%), *AKT1* (5.4% vs 3.9%), *ESR1* (3.7% vs 3.9%), *PTEN* (2.6% vs 3.2%), *PALB2* (1.5% vs 0.7%), and *PD-L1* amplification (0.6% vs 0.7%). Among HER2 + stage I-III (*N* = 95) and stage IV (*N* = 78) breast cancers, the most frequent alterations, other than *HER2* amplification, were *PIK3CA* mutations (26% vs 41.0%), followed by *BRCA1/2* (6.3% vs 6.4%), *ESR1* (1.1% vs 3.9%) mutations, and *PD-L1* amplification (1.1% vs 0%). Among TNBC stage I-III (*N* = 321) and stage IV (*N* = 216) breast cancers, the most frequent alterations were mutations in *PIK3CA* (19.9% vs 21.3%), *BRCA1/2* (11.5% vs 12.0%), *ESR1* (0.31% vs 0%), *PALB2* (0.9% vs 1.4%), and *PD-L1* amplification (2.2% vs 4.6%).

Across all cancer-related genes that were assessed, only *RAD21* mutations (currently not considered actionable/targetable with approved drugs) had significantly higher prevalence in stage IV HR+/HER2− tumors compared to stage I-III disease (18.4% *vs* 9.7%; FDR = 0.04; OR 2.1, 95% CI: 1.4-3.2; Supplementary Figure 1). No statistically significant differences were found in any genomic alterations in the other receptor subtypes.

### Targetable biomarkers in stage I-III cancers with early and late relapse

Among stage I-III cancers, 515 had a known recurrence date and included 260 cancers with early and 255 with late recurrence. When comparing patients with late versus early recurrence, those with late recurrence were statistically significantly more likely to have presented with stage I disease (20% vs 10.8%, *P* = .02), less likely to have stage III disease (33.3% vs 48.8%, *P* = .003), and were more frequently HR+/HER2- (66% vs 40.1%, *P* < .001) and less frequently TNBC (26.1% vs 52.1%, *P* < .001; Table 2). No statistical differences were found in any other clinical and molecular features.

**Table 2.** Study population and the clinicopathologic characteristics of specimens of stage I-III cancers with early (<2 years after the diagnosis) or late recurrence (≥2 years after the diagnosis).

Clinicopathologic characteristics	Early recurrence (*N* = 260)	Late recurrence (*N* = 255)	*P* val (FDR)
*Age at diagnosis*			.15
Median (Q1, Q3)	57 (49, 69)	55 (46, 65)	
*Group stage*			
I	28 (10.8%)	51 (20.0%)	.02
II	105 (40.4%)	119 (46.7%)	.27
III	127 (48.8%)	85 (33.3%)	.003
*Histology*			
IDC	209 (80.4%)	200 (78.4%)	.71
ILC	25 (9.6%)	42 (16.5%)	.08
Other	21 (8.1%)	8 (3.1%)	.08
Unknown/not documented	5 (1.9%)	5 (2.0%)	1
*HRDsig*			.69
positive	53 (20.4%)	46 (18.0%)	
negative	207 (79.6%)	209 (82.0%)	
*TMB*			.23
<10	246 (94.6%)	249 (97.6%)	
≥10	14 (5.4%)	6 (2.4%)	
*MSI-H*			.69
MSI I/L	258 (99.2%)	255 (100.0%)	
MSI-H	2 (0.8%)	0 (0.0%)	
*Receptor status*			
HER2+	20 (7.8%)	20 (7.9%)	1
HR+/HER2-	103 (40.1%)	167 (66.0%)	<.001
TNBC	134 (52.1%)	66 (26.1%)	<.001
N-Miss	3	2	
*Predicted ancestry*			
AFR	36 (13.8%)	20 (7.8%)	.09
AMR	20 (7.7%)	23 (9.0%)	.71
EAS	6 (2.3%)	3 (1.2%)	.69
EUR	198 (76.2%)	209 (82.0%)	.25
SAS	0	0	
*PD-L1 status (DAKO 22C3), CPS ≥ 10*			.71
Positive	6 (60.0%)	3 (37.5%)	
Negative	4 (40.0%)	5 (62.5%)	
N-Miss	250	247	

Abbreviations: *P* val, *P*-value; FDR, false discovery rate; Q1, 1st quartile; Q3, 3rd quartile; AFR, Africans; AMR, Admixed Americans; EAS, East Asians; EUR, Europeans; SAS, South Asians; PD-L1, programmed cell death ligand 1; CPS, combined positive score; N-Miss, data missing; TMB, tumor mutational burden; MSI-H, microsatellite instability-high; MSI I/L, microsatellite instability- indeterminate/low; HR, hormone receptor; HER2, human epidermal growth factor receptor 2; TNBC, triple-negative breast cancer; IDC, invasive ductal carcinoma; ILC, invasive lobular carcinoma.

Similarly, no statistical differences were seen in currently targetable alterations between the early and late recurrence groups (Figure 2B). In the HR+/HER2- stage I-III breast cancers with early (*N* = 103) and late (*N* = 167) recurrence, the most frequent alteration was *PIK3CA* mutation (39.8% vs 45.5%), followed by *BRCA1/2* (11.7% vs 7.2%), *AKT1* (7.8% vs 4.8%), *ESR1* (3.9% vs 4.8%), *PTEN* (2.9% vs 2.4%), *PALB2* (1.0% vs 1.2%), and *PD-L1* amplification (0% vs 0.6%). Among HER2 + stage I-III breast cancers with early (*N* = 20) and late (*N* = 20) recurrence, the most frequent alteration, other than HER2 amplification, was *PIK3CA* (45% vs 25%), followed by *BRCA1/2* (10% vs 5%), *ESR1* (0% vs 5%), *PD-L1* amplification (5% vs 0%). Among TNBC stage I-III breast cancers with early (*N* = 134) and late (*N* = 66) recurrence, the most frequent alteration was *PIK3CA* (20.2% vs 33.3%), followed by *BRCA1/2* (6.0% vs 9.1%), *PD-L1* amplification (2.2% vs 3.0%), *PALB2* (1.5% vs 0%), and *ESR1* (0% vs 1.5%).

There were no significant differences in the frequency of targetable alterations between European and African ancestry groups in any of the 3 receptor subtypes, or by clinical stage, or early versus late recurrence in stage I-III cancers (Supplementary Table 1-4).

### Prevalence of HRR alterations and HRDsig

**Figure 3.**
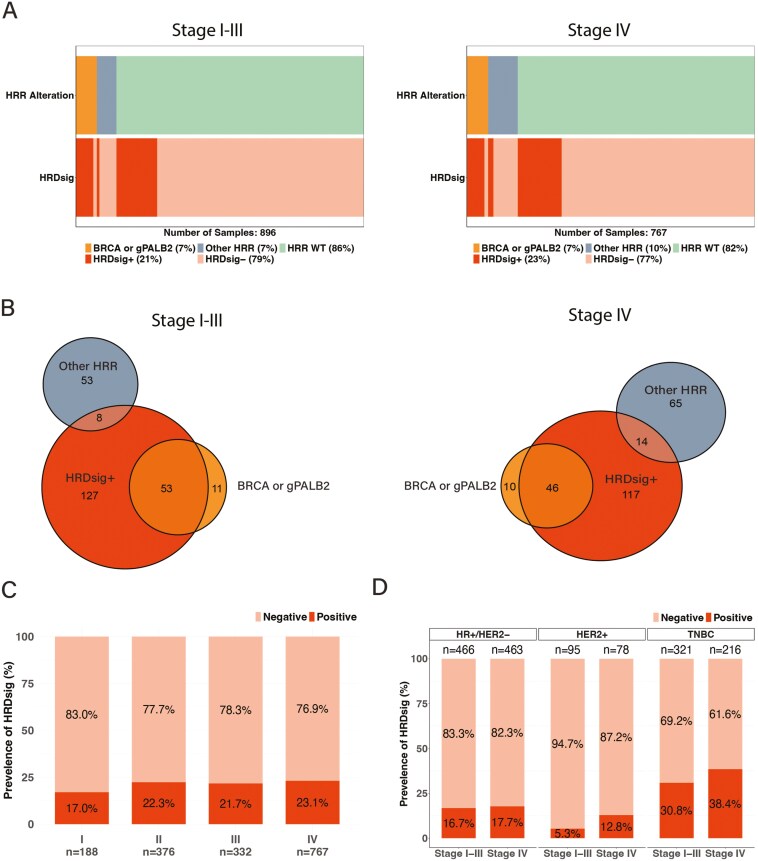
Prevalence of HRR alterations and HRDsig in stage I-III and stage IV breast cancers. **(A)** HRDsig positivity in cancers with altered g/sBRCA/gPALB, other known HRR genes, and HRR WT in stage I-III and stage IV breast cancer (orange = alterations in *BRCA* (genomic and somatic) and *gPALB2* (genomic PALB2), gray = alterations in other HRR genes (*ATM, BARD1, BRIP1, CDK12, CHEK1, CHEK2, FANCL, sPALB2, RAD51B, RAD51C, RAD51D* and *RAD54L*), green = HRR WT, red = HRDsig positive, light orange = HRDsig negative). **(B)** Venn diagram of HRDsig positivity in cancers with alterations in g/s*BRCA*/gPALB or other HRR genes in stage I-III and stage IV breast cancers. **(C)** Prevalence of HRDsig positivity in stage I, II, III, and IV cancers. **(D)** HRDsig positivity rates in stage I-III and IV cancers and hormone receptor subtypes. Abbreviations: HRDsig, homologous recombination deficiency signature, HRR, homologous recombination repair, WT, wild type, g/s, germline or/and somatic mutation, *BRCA,* germline or somatic breast cancer gene 1 and 2; *PALB2,* partner and localizer of *BRCA2; ATM,* ataxia-telangiectasia mutated; *BARD1, BRCA1* associated ring domain 1; *BRIP1, BRCA1* interacting protein 1; *CDK12,* cyclin-dependent kinase 12; *CHEK1,* checkpoint kinase 1; *CHEK2,* checkpoint kinase 2; *FANCL,* Fanconi anemia complementation group; *RAD51B,* RAD51 Paralog B; *RAD51C,* RAD51C Paralog C; *RAD51D,* RAD51C Paralog D; *RAD54L,* RAD54 Like.

In stage I-III cancers, the frequency of germline or somatic *g/sBRCA* mutation or *gPALB2* alteration that qualifies for PARPi therapy in the metastatic setting was 7% (64/896). The frequency of other HRR gene alterations (*ATM, BARD1, BRIP1, CDK12, CHEK1, CHEK2, FANCL, sPALB2, RAD51B, RAD51C, RAD51D,* and *RAD54*L) was also 7% (61/896). The remaining 86% (771/896) were wild type for all HRR genes. In stage IV cancers, the corresponding frequencies were similar, 7% (56/767), 10% (79/767), and 82% (632/767), respectively (Figure 3A). The frequencies of HRDsig positivity were also similar between the stage I-III and IV cancers (21% vs 23.1%).

Among *g/sBRCA* / g*PALB2* mutant cancers HRDsig positivity was 82% (*N* = 53/64) and 82.1% (*N* = 46/56), in stage I-III and IV cancers, respectively. Among stage I-III breast cancers with biallelic *g/sBRCA1/2/gPALB2* mutations, 98% (49/50) were HRDsig positive. Among cancers with other HRR gene alterations, HRDsig positivity rates for stage I-III and stage IV cancers were 13.1% (8/61) and 17.7% (14/79), respectively, not significantly different. Among HRR wild-type cancers, these frequencies were 16.5% in stage I-III (127/771) and 18.5% (117/632) in stage IV (Figure 3B). No statistical difference was found in HRDsig positivity when stage I (17% [32/188]), stage II (22.3% [84/376]), stage III (21.7% [72/332]), and stage IV (23.1% [177/767]) cancers were compared (*P* = .31; Figure 3C). HRDsig positivity was also analyzed in the stage I-III and stage IV breast cancers after stratification by HR receptor subtypes (Figure 3D). HRDsig positivity was most prevalent in stage I-III (*N* = 321) and IV (*N* = 216) TNBC 30.8% and 38.4%, respectively, followed by HR+/HER2− stage I-III (*N* = 466) and IV (*N* = 463) cancers 16.7% vs 17.7%, and HER2+ stage I-III (*N* = 95) and IV (*N* = 78) cancers 5.3% and12.8%. These HRDsig positivity rates were not statistically significantly different across stages.

## Discussion

In this study, we investigated genomic alterations in tissue biopsies of stage I-III and *de novo* stage IV breast cancers that underwent routine genomic testing within 3 months of diagnosis. We compared the frequencies of genomic alterations in over 300 cancer-related genes and a novel HRDsig biomarker between stage I-III and stage IV cancers and also between stage I-III breast cancers with early and late relapse. Overall, stage IV cancers were more frequently HR-positive (61.2% vs 52.8%, *P* = .005), and less frequently TNBC (28.5% vs 36.4%, *P* = .005) than stage I-III cancers. Within the same hormone receptor subtype, stage I-III and IV cancers had similar prevalence of targetable genomic alterations and HRDsig positivity.

PIK3CA mutation was the most prevalent currently actionable mutation in all clinical stages affecting around 40% of cancers. Genomic analysis of tumor biopsies at diagnosis can reveal persistent actionable mutations that could guide therapy at recurrence. For *de novo* stage IV HR+ /HER2- breast cancers with PIK3CA mutation, inavolisib plus a CDK4/6 inhibitor and endocrine therapy is now the standard of care.^21^ We also observed relatively frequent PD-L1 amplification in TNBC, especially among de novo stage IV TNBC (4.6%). A randomized Phase II trial has demonstrated the efficacy of anti-PD-L1 therapy in PD-L1 amplified cancers.^22^

Across all assessed genes, the mutation landscapes of stage I-III and stage IV cancers were highly similar with the exception of more frequent alterations in *RAD21* in stage IV HR+/HER2− cancers. *RAD21* mutations are not actionable currently but these alterations may contribute to generating genetic diversity via impaired DNA double-strand break repair and chromosomal segregation leading to higher metastatic potential,^23^ which could explain the distant metastases at the time of diagnosis. No difference in mutation frequencies in the primary tumor across stage I-III breast cancers has been shown previously,^24^ our study extends this observation to de novo stage IV cancers. Genomic alteration patterns and gene expression profiles of the primary tumor are primarily determined by molecular subtype and not by stage at presentation.

We also found that stage I-III cancers with early metastatic recurrence had similar genomic alterations in the primary tumor as those who had late recurrence. The prevalence of targetable alterations was different by hormone receptor subtype but not by early versus late recurrence. Luen et al. have evaluated the genomic driver alteration associated with distant recurrences ≥5 years in postmenopausal HR+/HER2- breast cancers.^25^ They found that those with 8p11 and *BRCA2* mutations had a higher risk of late distant recurrence compared to wild-type cancers. They also observed a lower risk of late recurrence with *PIK3CA* mutations in univariable analysis (but not in multivariable analysis). In TNBC patients, Zhang et al. have reported no statistical differences in TMB or percent genome alteration in early (≤2 years) versus late/no recurrence groups.^18^ In our study, we found no significant difference in targetable mutations in early versus late recurrence groups within receptor subtypes but noted that TNBC and stage III cancers tended to recur early while HR+ HER2− cancers and stage I disease tended to recur late. Clinical risk factors and transcriptomic features of the primary tumor appear to be more predictive of late recurrence than particular mutations.^24,26^

HRDsig is a novel biomarker that was developed to detect genomic scars indicative of HRD from genomic, epigenetic, or transcriptional dysfunctions and to identify patients who may benefit from PARP inhibitors and/or platinum drugs. HRDsig has been assessed in ovarian, breast, pancreatic, and prostate cancers and preliminary results suggest that HRDsig-positive tumors could benefit from PARP inhibitor and/or platinum therapy.^27-30^ In our study, approximately 20% of breast cancers across all stages were HRDsig positive. The highest prevalence was seen in TNBC ~30%, followed by ~17% in ER+/HER2− cancers, and ~9% of HER2+ cancers. HRDsig positivity was seen in 82% of breast cancers with g/s*BRCA1/2* or g*PALB2* mutation. HRDsig positivity was also detected in 13.1% of cancers with alterations in HRR genes other than *BRCA1/2*, and 16.5% of breast cancers wild type for all HRR genes were also HRDsig positive. A randomized Phase II trial, recently demonstrated that the addition of veliparib to cisplatin significantly improved progression-free survival in patients with BRCA-like metastatic triple-negative breast cancer, but not in patients with non-BRCA-like metastatic breast cancer.^31^ Considering that only 120 patients in our study had a *BRCA1/2* or g*PALB2* alteration, HRDsig was able to identify an additional 245 patients who might benefit from PARP inhibitors or platinum-based treatments. This hypothesis can be tested in the clinic.

In this study, we only analyzed specimens that were obtained within 3 months of diagnosis in order to be able to compare the de novo stage IV disease with stage I-III cancers without the confounding effects of prior therapy. It is possible that prior treatment status impacts actionable mutations and HRDsig, that can be evaluated in future studies.

In summary, our study shows that primary tumors of stage I-III and stage IV breast cancers share similar targetable genetic alterations and similar overall genomic landscapes. The genomic landscape of stage I-III breast cancers with early versus late recurrence is also similar. We show that approximately 20% of breast cancers have HRD captured by the HRDsig. This raises the possibility that HRDsig positive breast cancers that currently do not qualify for PARP inhibitors because they lack germline *g/sBRCA* and *gPALB2* alterations might benefit from PARP inhibitors and platinum-based treatments. However, the treatment predictive value of HRDsig in breast cancer patients will need to be validated in future clinical trials.

## Supplementary Material

oyaf089_suppl_Supplementary_Figures_1

oyaf089_suppl_Supplementary_Tables_1-4

## Data Availability

The data that support the findings of this study originated from Flatiron Health, Inc. and Foundation Medicine, Inc. Requests for data sharing by license or by permission for the specific purpose of replicating results in this manuscript can be submitted to PublicationsDataaccess@flatiron.com and cgdb-fmi@flatiron.com.
