# Supplementary material for: Genomic landscape and homologous recombination repair deficiency signature in stage I-III and de novo stage IV primary breast cancers
Source: Oncologist. 2025 May 27;30(5):oyaf089. doi: 10.1093/oncolo/oyaf089 (PMC12107548; doi:10.1093/oncolo/oyaf089)
Supplement: oyaf089_suppl_Supplementary_Figures_1 [file oyaf089_suppl_supplementary_figures_1.docx]

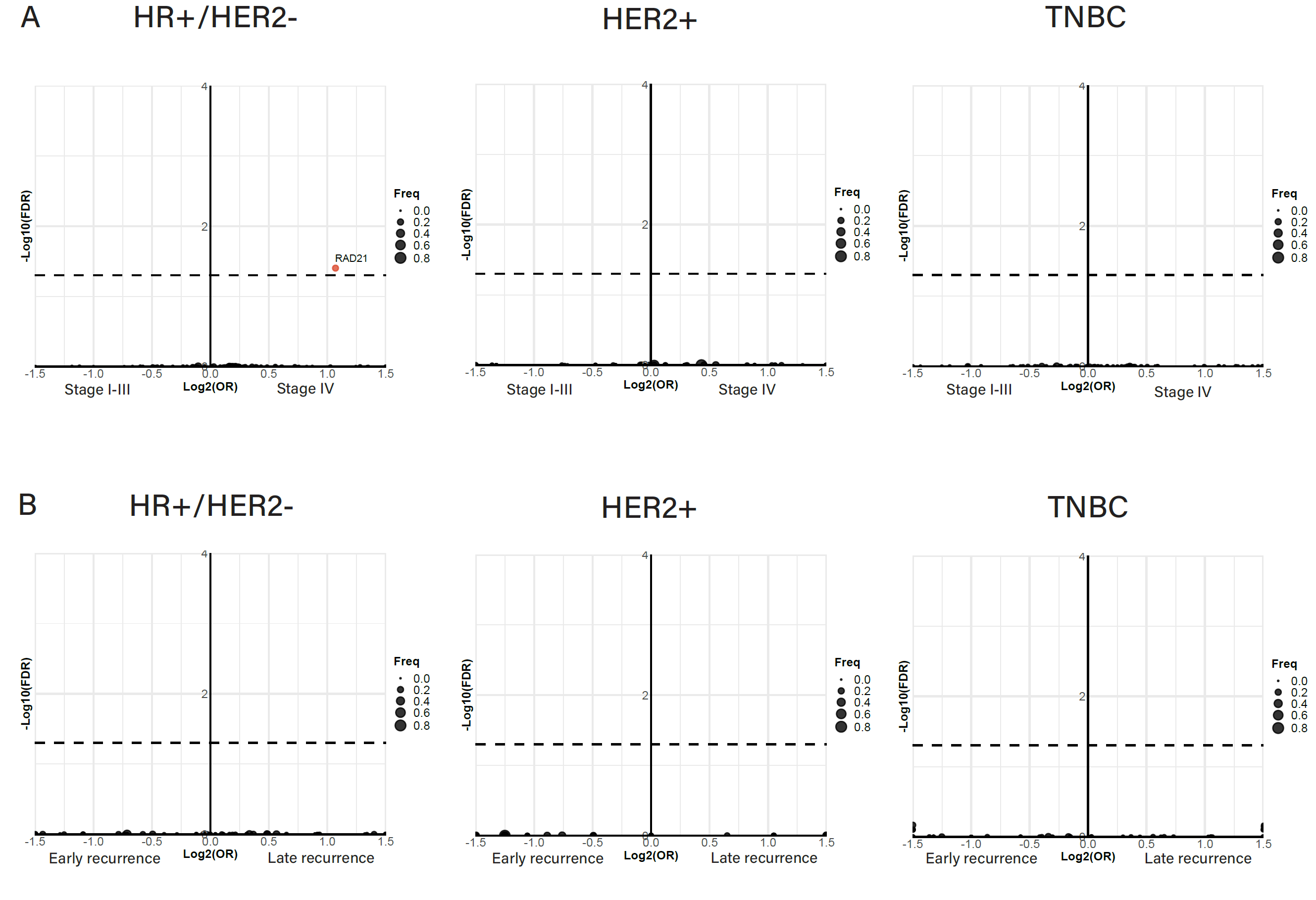


**Supplemental Figure 1.** Volcano plots of 324 genes with genomic alterations in three receptor subtypes (HR+/HER2-, HER2+, TNBC). **A,** comparison between stage I-III versus de novo stage IV. *RAD21* gene had significantly higher alteration frequency (freq = 0.18, FDR = 0.04) with odds ratio of 2.1 in favor of the de novo stage IV in the HR+/HER2- group. **B,** comparison between early versus late recurrence among the stage I-III group. No statistically significant difference in gene alterations frequencies were found between the early and late recurrence groups. X axis is represented as Log2 of the odds ratio and Y axis is represented as -log10 of the FDR. The dotted horizontal line represents the significance threshold of Benjamini-Hochberg false discovery rate (FDR) = 0.05. The size of the dot is proportional to the frequency of the alterations. Abbreviations: HR, hormone receptor; HER2, human epidermal growth factor receptor 2; TNBC, triple negative breast cancer; freq, gene alteration frequency; OR, odds ratio; Log2(OR), log2 of the odds ratio; FDR, false discovery rate; -Log10(FDR), negative log10 of the false discovery rate.
