# Supplementary material for: Genomic landscape and homologous recombination repair deficiency signature in stage I-III and de novo stage IV primary breast cancers
Source: Oncologist. 2025 May 27;30(5):oyaf089. doi: 10.1093/oncolo/oyaf089 (PMC12107548; doi:10.1093/oncolo/oyaf089)
Supplement: oyaf089_suppl_Supplementary_Tables_1-4 [file oyaf089_suppl_supplementary_tables_1-4.docx]

| **Stage I-III** | **HR+/HER2-** | | | **HER2+** | | | **TNBC** | | |
| --- | --- | --- | --- | --- | --- | --- | --- | --- | --- |
|  | **EUR (N = 342)** | **AFR (N = 58)** |  | **EUR (N = 63)** | **AFR (N = 13)** |  | **EUR (N = 213)** | **AFR (N = 57)** |  |
|  | **Prevelence of alteration(%)** | **Prevelence of alteration(%)** | **P val (FDR)** | **Prevelence of alteration(%)** | **Prevelence of alteration(%)** | **P val (FDR)** | **Prevelence of alteration(%)** | **Prevelence of alteration(%)** | **P val (FDR)** |
| HRDsig | 16.08 | 20.69 | 0.97 | 3.17 | 15.38 | 1 | 28.17 | 26.32 | 1 |
| PIK3CA mut | 42.69 | 37.93 | 0.97 | 30.16 | 23.08 | 1 | 23.47 | 10.53 | 0.38 |
| ESR1 mut | 3.8 | 1.72 | 0.97 | 1.59 | 0 | 1 | 0.47 | 0 | 1 |
| BRCA1/2 alt | 8.48 | 8.62 | 1 | 6.35 | 7.69 | 1 | 7.98 | 12.28 | 0.68 |
| PALB2 mut | 0.88 | 1.72 | 0.97 | 0 | 0 | 1 | 1.41 | 0 | 1 |
| PDL1 amp | 0.58 | 1.72 | 0.97 | 1.59 | 0 | 1 | 2.82 | 1.75 | 1 |
| NTRK fusion | 0 | 0 | 1 | 0 | 0 | 1 | 0 | 0 | 1 |
| AKT1 mut | 5.56 | 3.45 | 0.97 | 4.76 | 0 | 1 | 3.76 | 0 | 0.68 |
| PTEN loss | 2.05 | 3.45 | 0.97 | 0 | 0 | 1 | 7.04 | 12.28 | 0.68 |

**Supplemental Table 1.** Comparison of the frequency of targetable biomarkers between European and African groups using Fisher’s exact test across different receptor subtypes within the stage I-III group. P-values were adjusted for multiple comparisons using the FDR method within each receptor status. Abbreviations: HR, hormone receptor; HER2, human epidermal growth factor receptor 2; TNBC, triple negative breast cancer; EUR, European ancestry; AFR, African ancestry; P val, p-value; FDR, false discovery rate; HRDsig, homologous recombination deficiency signature; *PIK3CA* mut, phosphatidylinositol-4,5-bisphosphate 3-kinase catalytic subunit alpha gene mutation; *BRCA 1/2* alt, breast cancer gene 1 and 2 alteration; *ESR1* mut, estrogen receptor alpha gene mutation; *PDL1* amp, programmed cell death ligand 1 gene amplification; *PALB2* mut, partner and localizer of *BRCA2* gene mutation; *NTRK* fusion, neurotrophic tyrosine receptor kinase gene fusion, *AKT1* mut: RAC-alpha serine/threonine-protein kinase gene mutation; *PTEN* loss: phosphatase and tensin homolog gene loss.

| **Stage IV** | **HR+/HER2-** | | | **HER2+** | | | **TNBC** | | |
| --- | --- | --- | --- | --- | --- | --- | --- | --- | --- |
|  | **EUR (N = 364)** | **AFR (N = 57)** |  | **EUR (N = 52)** | **AFR (N = 12)** |  | **EUR (N = 139)** | **AFR (N = 47)** |  |
|  | **Prevalence of alteration (%)** | **Prevalence of alteration (%)** | **P val (FDR)** | **Prevalence of alteration (%)** | **Prevalence of alteration (%)** | **P val (FDR)** | **Prevalence of alteration (%)** | **Prevalence of alteration (%)** | **P val (FDR)** |
| HRDsig | 17.58 | 21.05 | 0.87 | 13.46 | 8.33 | 1 | 41.73 | 27.66 | 0.87 |
| PIK3CA mut | 43.96 | 38.6 | 0.86 | 44.23 | 41.67 | 1 | 21.58 | 19.15 | 1 |
| ESR1 mut | 4.67 | 0 | 0.65 | 3.85 | 0 | 1 | 0 | 0 | 1 |
| BRCA1/2 alt | 7.42 | 8.77 | 1 | 7.69 | 8.33 | 1 | 11.51 | 10.64 | 1 |
| PALB2 mut | 0.27 | 3.51 | 0.44 | 0 | 0 | 1 | 0.72 | 2.13 | 1 |
| PDL1 amp | 0.55 | 1.75 | 0.8 | 0 | 0 | 1 | 5.04 | 4.26 | 1 |
| NTRK fusion | 0 | 0 | 1 | 0 | 0 | 1 | 0 | 0 | 1 |
| AKT1 mut | 4.4 | 3.51 | 1 | 0 | 0 | 1 | 2.16 | 2.13 | 1 |
| PTEN loss | 3.85 | 0 | 0.7 | 0 | 0 | 1 | 5.04 | 0 | 0.87 |

**Supplemental Table 2.** Comparison of the frequency of targetable biomarkers between European and African groups using Fisher’s exact test across different receptor subtypes within the stage IV group. P-values were adjusted for multiple comparisons using the FDR method within each receptor status. Abbreviations: HR, hormone receptor; HER2, human epidermal growth factor receptor 2; TNBC, triple negative breast cancer; EUR, European ancestry; AFR, African ancestry; P val, p-value; FDR, false discovery rate; HRDsig, homologous recombination deficiency signature; *PIK3CA* mut, phosphatidylinositol-4,5-bisphosphate 3-kinase catalytic subunit alpha gene mutation; *BRCA 1/2* alt, breast cancer gene 1 and 2 alteration; *ESR1* mut, estrogen receptor alpha gene mutation; *PDL1* amp, programmed cell death ligand 1 gene amplification; *PALB2* mut, partner and localizer of *BRCA2* gene mutation; *NTRK* fusion, neurotrophic tyrosine receptor kinase gene fusion, *AKT1* mut: RAC-alpha serine/threonine-protein kinase gene mutation; *PTEN* loss: phosphatase and tensin homolog gene loss.

| **Early recurrence** | **HR+/HER2-** | | | **HER2+** | | | **TNBC** | | |
| --- | --- | --- | --- | --- | --- | --- | --- | --- | --- |
|  | **EUR (N = 80)** | **AFR (N = 10)** |  | **EUR (N = 15)** | **AFR (N = 4)** |  | **EUR (N = 100)** | **AFR (N = 22)** |  |
|  | **Prevalence of alteration (%)** | **Prevalence of alteration (%)** | **P val (FDR)** | **Prevalence of alteration (%)** | **Prevalence of alteration (%)** | **P val (FDR)** | **Prevalence of alteration(%)** | **Prevalence of alteration (%)** | **P val (FDR)** |
| HRDsig | 20 | 30 | 1 | 0 | 25 | 1 | 19 | 18.18 | 1 |
| PIK3CA mut | 40 | 20 | 1 | 46.67 | 50 | 1 | 23 | 13.64 | 1 |
| ESR1 mut | 2.5 | 10 | 1 | 0 | 0 | 1 | 0 | 0 | 1 |
| BRCA1/2 alt | 12.5 | 10 | 1 | 6.67 | 25 | 1 | 3 | 9.09 | 1 |
| PALB2 mut | 0 | 0 | 1 | 0 | 0 | 1 | 2 | 0 | 1 |
| PDL1 amp | 0 | 0 | 1 | 6.67 | 0 | 1 | 3 | 0 | 1 |
| NTRK fusion | 0 | 0 | 1 | 0 | 0 | 1 | 0 | 0 | 1 |
| AKT1 mut | 7.5 | 10 | 1 | 0 | 0 | 1 | 4 | 0 | 1 |
| PTEN loss | 2.5 | 0 | 1 | 0 | 0 | 1 | 7 | 13.64 | 1 |

**Supplemental Table 3.** Comparison of the frequency of targetable biomarkers between European and African groups using Fisher’s exact test across different receptor subtypes within the early recurrence group. P-values were adjusted for multiple comparisons using the FDR method within each receptor status. Abbreviations: HR, hormone receptor; HER2, human epidermal growth factor receptor 2; TNBC, triple negative breast cancer; EUR, European ancestry; AFR, African ancestry; P val, p-value; FDR, false discovery rate; HRDsig, homologous recombination deficiency signature; *PIK3CA* mut, phosphatidylinositol-4,5-bisphosphate 3-kinase catalytic subunit alpha gene mutation; *BRCA 1/2* alt, breast cancer gene 1 and 2 alteration; *ESR1* mut, estrogen receptor alpha gene mutation; *PDL1* amp, programmed cell death ligand 1 gene amplification; *PALB2* mut, partner and localizer of *BRCA2* gene mutation; *NTRK* fusion, neurotrophic tyrosine receptor kinase gene fusion, *AKT1* mut: RAC-alpha serine/threonine-protein kinase gene mutation; *PTEN* loss: phosphatase and tensin homolog gene loss.

| **Late recurrence** | **HR+/HER2-** | | | **HER2+** | | | **TNBC** | | |
| --- | --- | --- | --- | --- | --- | --- | --- | --- | --- |
|  | **EUR (N = 140)** | **AFR (N = 11)** |  | **EUR (N = 16)** | **AFR (N = 1)** |  | **EUR (N = 51)** | **AFR (N = 8)** |  |
|  | **Prevalence of alteration (%)** | **Prevalence of alteration (%)** | **P val (FDR)** | **Prevalence of alteration (%)** | **Prevalence of alteration (%)** | **P val (FDR)** | **Prevalence of alteration (%)** | **Prevalence of alteration (%)** | **P val (FDR)** |
| HRDsig | 13.57 | 27.27 | 0.61 | 0 | 0 | 1 | 31.37 | 25 | 1 |
| PIK3CA mut | 46.43 | 27.27 | 0.78 | 31.25 | 0 | 1 | 33.33 | 37.5 | 1 |
| ESR1 mut | 5 | 0 | 1 | 6.25 | 0 | 1 | 1.96 | 0 | 1 |
| BRCA1/2 alt | 6.43 | 18.18 | 0.61 | 6.25 | 0 | 1 | 9.8 | 12.5 | 1 |
| PALB2 mut | 0 | 9.09 | 0.61 | 0 | 0 | 1 | 0 | 0 | 1 |
| PDL1 amp | 0.71 | 0 | 1 | 0 | 0 | 1 | 3.92 | 0 | 1 |
| NTRK fusion | 0 | 0 | 1 | 0 | 0 | 1 | 0 | 0 | 1 |
| AKT1 mut | 5 | 0 | 1 | 18.75 | 0 | 1 | 5.88 | 0 | 1 |
| PTEN loss | 2.14 | 0 | 1 | 0 | 0 | 1 | 9.8 | 25 | 1 |

**Supplemental Table 4.** Comparison of the frequency of targetable biomarkers between European and African groups using Fisher’s exact test across different receptor subtypes within the late recurrence group. P-values were adjusted for multiple comparisons using the FDR method within each receptor status. Abbreviations: HR, hormone receptor; HER2, human epidermal growth factor receptor 2; TNBC, triple negative breast cancer; EUR, European ancestry; AFR, African ancestry; P val, p-value; FDR, false discovery rate; HRDsig, homologous recombination deficiency signature; *PIK3CA* mut, phosphatidylinositol-4,5-bisphosphate 3-kinase catalytic subunit alpha gene mutation; *BRCA 1/2* alt, breast cancer gene 1 and 2 alteration; *ESR1* mut, estrogen receptor alpha gene mutation; *PDL1* amp, programmed cell death ligand 1 gene amplification; *PALB2* mut, partner and localizer of *BRCA2* gene mutation; *NTRK* fusion, neurotrophic tyrosine receptor kinase gene fusion, *AKT1* mut: RAC-alpha serine/threonine-protein kinase gene mutation; *PTEN* loss: phosphatase and tensin homolog gene loss.
